# Biochemical engineering provides mindset, tools and solutions for the driving questions of a sustainable future

**DOI:** 10.1002/elsc.201900150

**Published:** 2019-11-12

**Authors:** Ralf Takors

**Affiliations:** ^1^ Institute of Biochemical Engineering University of Stuttgart Stuttgart Germany

In 2015, United Nations (UN) released 17 sustainability goals as part of the 2030 Agenda for ensuring sustainable development worldwide. In December of the same year, an overwhelming majority of nations agreed to join the Paris Climate Agreement putting the reduction of greenhouse gas release in the center of future actions. ‘*Fridays for Future*’ is transferring the academic findings and political resolutions in a public movement, now pushing public opinion, industrial and political decision makers further. Clearly, environmental protection, prevention of further climate change, and sustainable development are no longer ‘*nice‐to‐have*’ options, they are getting essential parts of our daily life asking for solutions on many fields including food, farming, health, and industrial production.

Accordingly, biochemical engineers need to wonder: What is our part in the ongoing structural change? The answer is: It is an essential role that biochemical engineers have to play!

A glimpse on only a few UN goals explains how important biochemical engineering can be to reach the aims.

#2′ *Zero Hunger*’: The worldwide population is steadily increasing, counting 7.7 billion in August 2019. Numerous scenarios predict a shortage of protein because the conventional way of animal farming will reach ethical and environmental limits, soon. Developments such as the ‘*Impossible Burger*’ may be one alternative making use of recombinant heme (soy leghemoglobin) produced in yeast. Insect farming may be another option. Compared to the conventional production of animal protein, greenhouse gas emissions are significantly lower and production can happen on ‘*waste streams*’ not requiring for highly valuable farm land. Irrespective what option will dominate in the future, it should be the role of biochemical engineering to support the step from the lab to large‐scale application. Notably, non‐conventional protein production intrinsically also opens the door for alternate use not only in food but also in feed markets and for the exploitation of side streams to get high value products.

#3 ‘*Good Health and Well‐Being*’: Pharmaceutical industry currently faces major changes. Budgets of public health systems in the developed world are getting limiting, more and more, while novel diseases and novel therapies ask for new solutions and new applications. Additionally, market weights are likely to change, e.g. putting African nations and their ‘*forgotten’* diseases much more in the focus of drug development. Accordingly, low‐cost, flexible biopharmaceutical production processes are needed to serve the rising demands, also paving the way to a personalized medicine. Apparently, biochemical engineers should offer technical solutions for low‐cost production of biopharmaceuticals, also considering small‐scale on demand production devices.

#9, 12, 13 ‘*Industry Innovation & Infrastructure*’, ‘*Responsible Consumption & Production*’, ‘*Climate Action*’: Basically, the three goals are standing on common ground. They ask for the establishment of a circular economy, ideally eliminating waste completely which comprises the re‐use of CO_2_, too. The latter requires the activation of CO_2_ via electrons which creates links to electro‐, chemical, and phototrophic applications where single strains of distinct species such as acetogens or even well composed consortia may convert the gas to commodities and fine chemicals. Besides, biochemical engineering is expected to enable the access to novel or already existing products using other sustainable resources such as (lignocellulosic‐) sugars. To deal with existing infrastructure and markets those processes need to find the way from the lab to industrial, large‐scale application.

For sure, the above list is far from being complete. For instance, research activities aiming to produce plant products prevent ongoing deforestation because mono‐culture plantation may have served for the same job instead (#15). Furthermore, classical research activities of water, soil and air purification will continue to develop novel approaches, still in line with the current UN sustainability agenda.

Summarizing, biochemical engineering has a lot to offer and provides numerous solutions for today's most driving questions. Now, there is the time to make use of the ideas, to exploit their potential.

In its infancy, biotechnology succeeded creating products that could not be accessed by conventional approaches. Later on, technologies matured and even replaced existing routes when economic market constraints were achieved. Now, we may enter a new age of biotechnological applications, mainly driven by ecological demands. Biochemical engineers are called to pave the way for novel sustainable processes in accordance with ecological and economical needs!

